# Basic Research in Endocrinology: A Modern Strategy for the Development and Technologies of Personalized Medicine

**DOI:** 10.3390/jpm11090895

**Published:** 2021-09-08

**Authors:** Elena Shakhtshneider, Alla Ovsyannikova, Oksana Rymar, Yuliya Ragino, Mikhail Voevoda

**Affiliations:** 1 Institute of Internal and Preventive Medicine—Branch of Institute of Cytology and Genetics, Siberian Branch of Russian Academy of Sciences (SB RAS), 175/1 Borisa Bogatkova Str., Novosibirsk 630089, Russia; 2117409@mail.ru (E.S.); aknikolaeva@bk.ru (A.O.); orymar23@gmail.com (O.R.); 2 Institute of Cytology and Genetics, Siberian Branch of Russian Academy of Sciences (SB RAS), 10 Prospect Ak. Lavrentyeva, Novosibirsk 630090, Russia

The first all-Russia conference with international participation, “Basic Research in Endocrinology: A Modern Strategy for the Development and Technologies of Personalized Medicine”, was held in Novosibirsk on 26–27 November 2020. The purpose of this conference was to disseminate the latest basic and clinical findings in the fields of etiology, clinical characteristics, and modern diagnostics and treatments of endocrine disorders among various relevant specialists. The conference was intended for practicing endocrinologists, primary care physicians, medical geneticists, pediatric endocrinologists, pediatricians, and physician–scientists. The conference included plenary sessions, specialty sessions, satellite symposia, an open competition for young scientists, and the first-in-Russia educational course for physicians: “Maturity Onset Diabetes of the Young (MODY): Molecular Genetic Determinants and a Personalized Approach to Patient Management.” This Special Issue on “Basic Research in Endocrinology: A Modern Strategy for the Development and Technologies of Personalized Medicine” includes a review and 10 original studies about diabetes mellitus, hypothalamic norepinephrine, obesity, and osteoporosis.

Six of the special-issue articles, Samoilova et al. [1], Mustafina et al. [2], Ivanoshchuk et al. [3,4], Musina et al. [5], and Kruchinina et al. [6], focus on the detection and characterization of various types of diabetes mellitus. Samoilova et al. [1] evaluated the specificity of hippocampal spectroscopy for parameters of type 1 and type 2 diabetes mellitus (T1DM and T2DM) and cognitive dysfunction. The authors analyzed 65 T1DM patients with cognitive deficits and 20 T1DM patients without, as well as 75 T2DM patients with cognitive deficits and 20 T2DM patients without. They determined that patients with diabetes possessed an altered hippocampal metabolism, which may serve as an early predictive marker of neurometabolic changes. The main modifiable risk factors whose correction may slow down the progression of cognitive dysfunction were identified. Mustafina et al. [2] investigated the 14-year risk of T2DM and developed a risk score for T2DM in a Siberian cohort. A random population sample (males/females, 45–69 years old) was examined at baseline in 2003–2005 (Health, Alcohol, and Psychosocial Factors in Eastern Europe [HAPIEE] project, *n* = 9360, Novosibirsk) and re-examined in 2006–2008 and 2015–2017. After the exclusion of subjects with baseline T2DM, the final analysis included 7739 participants. In addition, secondary education, low physical activity, and a history of cardiovascular disease were significantly associated with T2DM in females. Ivanoshchuk et al. [3] researched the genetic characteristics of MODY. The authors analyzed 14 MODY genes in 178 patients with a MODY phenotype in Western Siberia. A multiplex ligation-dependent probe amplification analysis of DNA samples from 50 randomly selected patients without detectable mutations did not reveal large rearrangements in the MODY genes. In 38 patients (among them, 37% males) out of the 178, mutations were identified in *HNF4A*, *GCK*, *HNF1A*, and *ABCC8*. The authors of ref. [4] investigated the *APPL1* gene, which is associated with MODY 14. Thirteen variants were found in *APPL1*, three of which (rs79282761, rs138485817, and rs11544593) were located in exons. There were no statistically significant differences in the frequencies of rs11544593 alleles and genotypes between T2DM patients and the general population. In the MODY group, AG rs11544593 genotype carriers were significantly more frequent (AG vs. AA + GG: odds ratio 1.83, confidence interval 1.15–2.90, *p* = 0.011) compared with the control group. Musina et al. [5] established relations among inflammatory status, ferrokinetics, and lipid metabolism in patients with diabetes mellitus. The discovered relations among lipid profile indices, inflammatory status, and microalbuminuria confirmed the mutual influences of hyperlipidemia, inflammation, and nephropathy in diabetes patients. Their results justify the strategy involving early hypolipidemic therapy for patients with diabetes mellitus to prevent the development and progression of microvascular complications. Kruchinina et al. [6] investigated the feasibility of a differential diagnosis of degrees of rheological disturbances in patients with T2DM by dielectrophoresis of erythrocytes. The proposed experimental approach features a low invasiveness, high productivity, shorter duration, and vividness of the results. The method allows one to evaluate not only the local (renal and ocular) but also systemic status of microcirculation using more than 20 parameters of erythrocytes.

Redina et al. [7] addressed the role of hypothalamic norepinephrine in the activation of the sympathetic nervous system. They carried out genetic mapping by quantitative trait loci analysis and identified loci associated both with an increased hypothalamic norepinephrine concentration and with an increase of the heart weight in Inherited Stress-Induced Arterial Hypertension (ISIAH) rats, a model of the stress-sensitive type of arterial hypertension. The contribution to the development of heart hypertrophy in ISIAH rats is controlled by different genetic loci, one of which is associated with hypothalamic norepinephrine concentration (on chromosome 18) and the other correlating with high blood pressure (on chromosome 1).

Kiseleva et al. [8] investigated correlations between the parameters of classic clinical blood tests and the proteomic profiles of 104 lean and obese subjects. As a result, the authors compiled patterns of the proteins whose presence or absence allowed one to predict the weight of a patient fairly well. Ragino et al. [9] studied the blood cytokine/chemokine profile of 25–44 year old patients with early ischemic heart disease comorbid with abdominal obesity. Their findings related to Flt3 ligand, granulocyte macrophage–colony stimulating factor (GM-CSF), and interleukin 4 (IL-4) are consistent with the international literature. The results of that study are partly confirmative and partly hypothesis-generating.

Mazurenko et al. [10] examined the frequency of osteoporotic forearm fractures in postmenopausal women to assess their association with risk factors of chronic noncommunicable diseases. In the studied population sample of postmenopausal women, a high total cholesterol level and a history of smoking were cross-sectional determinants of osteoporotic forearm fractures, whereas the body–mass index was a protective factor against the risk of osteoporotic fractures.

Grigor’eva [11] wrote a review about the relation of gallstone disease, obesity, and the Firmicutes/Bacteroidetes ratio as a possible biomarker of gut dysbiosis. This review presents and summarizes recent findings of studies on the gut microbiota in patients with gallstone disease.

The articles in this special issue cover interesting topics in endocrinology that are also related to gastroenterology, genetics, hematology, and cardiology. The presented data expand our knowledge about basic endocrinology.
